# Supplementary material for: Knowledge and prevalence of common sexually transmitted infections among patients seeking care at selected health facilities in Southern Ghana
Source: PLOS Glob Public Health. 2024 Jul 1;4(7):e0003422. doi: 10.1371/journal.pgph.0003422 (PMC11216559; doi:10.1371/journal.pgph.0003422)
Supplement: S1 Questionnaire — (DOCX) [file pgph.0003422.s001.docx]

Patient Code:

QUESTIONNAIRE

**COMMON SEXUALLY TRANSMITTED INFECTIONS (GONORRHOEA, SYPHILIS AND CHLAMYDIA) IN HO MUNICIPALITY AND THEIR MANAGEMENT**

**SECTION A: DEMOGRAPHIC CHARACTERISTICS**

1. Gender: [ ] Male [ ] Female

2. Age: ……… (years)

3. Education: [ ] No education [ ] Primary Education

[ ] Secondary Education [ ] Tertiary Education

4. Employment: …………………

5. Marital Status: [ ] Married [ ] Single [ ] Divorced [ ] Widowed

6. Religion: [ ] Christian [ ] Muslim Other (Please specify):_____________

**SECTION B: KNOWLEDGE ON STI**

**NB: The answers have been marked with ‘X’**

7. Have you heard about STIs?

[ ] Yes [ ] No [ ] I do not know

8. Types of STIs (Please tick all that apply)

Syphilis [X] Yes [ ] No

Gonorrhea [X] Yes [ ] No

Chlamydia [X] Yes [ ] No

HIV [X] Yes [ ] No

Herpes [X] Yes [ ] No

9. Mode of transmission

Sexual Contact [X] Yes [ ] No

Holding of hands [ ] Yes [X] No

Blood transfusion [X] Yes [ ] No

Mother-to-child transmission [X] Yes [ ] No

Hugging [ ] Yes [X] No

Kissing [ X ] Yes [] No

10. Signs and symptoms

Discharge from the penis or vagina [X] Yes [ ] No

Sores or rashes on the genital area [X] Yes [ ] No

Abdominal pain [X] Yes [ ] No

Painful urination [X] Yes [ ] No

Pain during sex [X] Yes [ ] No

11. Complications of STIs

Infertility [X] Yes [ ] No

Cancer [X] Yes [ ] No

Ectopic pregnancy [X] Yes [ ] No

Low birth weight [X] Yes [ ] No

12. Prevention of STI

Use of condoms [X] Yes [ ] No

Abstinence [X] Yes [ ] No

Avoid sex with anyone who has genital sores, a rash or discharge [X] Yes [ ] No

Multiple sexual partners [X] Yes [ ] No

**SECTION C: PATIENT FACTORS**

13. Do you drink alcohol?

[ ] Yes [ ] No

14. Do you smoke?

[ ] Yes [ ] No

**SECTION D: SEXUAL BEHAVIOUR**

15. How many sexual partners do you have currently? .............................

16. Do you prefer to use a condom when you have sex?

[ ] Yes [ ] No [ ] I do not know

17. How often do you have sex in a week? .............................

18. How many relationships have been in for the past one (1) year? Include the current one.............................

**SECTION F: HEALTH SEEKING BEHAVIOUR**

19. Where do you prefer to seek treatment when you have any sexually transmitted infection?

__________________________________________________________________________

20. How long do the symptoms of the STI persist before you seek treatment?

____________________________________________________________

21. Which treatment do you prefer?

[ ] Herbal medicine [ ] Orthodox medicine [ ] Faith Healer

Other (Please specify):___________________

22. Do you always comply with the treatment given to you?

[ ] Yes [ ] No [ ] Sometimes

23. Do you sometimes self-medicate?

[ ] Yes [ ] No [ ] Never

24. What do you take if you answered ‘yes’ to Question 23?

________________________________________________

**MICROBIOLOGY WORKSHEET**

FACILITY:

| **Date** | **Patient**  **ID** | **Age** | **Sex** | **Gonorrhoea** | **Chlamydia** | **Syphilis** |
| --- | --- | --- | --- | --- | --- | --- |
|  |  |  |  |  |  |  |
|  |  |  |  |  |  |  |
|  |  |  |  |  |  |  |
|  |  |  |  |  |  |  |
|  |  |  |  |  |  |  |
|  |  |  |  |  |  |  |
|  |  |  |  |  |  |  |
|  |  |  |  |  |  |  |
|  |  |  |  |  |  |  |
|  |  |  |  |  |  |  |
|  |  |  |  |  |  |  |
|  |  |  |  |  |  |  |
|  |  |  |  |  |  |  |
|  |  |  |  |  |  |  |
|  |  |  |  |  |  |  |
|  |  |  |  |  |  |  |
|  |  |  |  |  |  |  |
|  |  |  |  |  |  |  |
