# Supplementary material for: Knowledge and prevalence of common sexually transmitted infections among patients seeking care at selected health facilities in Southern Ghana
Source: PLOS Glob Public Health. 2024 Jul 1;4(7):e0003422. doi: 10.1371/journal.pgph.0003422 (PMC11216559; doi:10.1371/journal.pgph.0003422)
Supplement: Attachment — Submitted filename: Response to Reviewers.docx [file pgph.0003422.s003.docx]

Department of Pharmacy Practice

Faculty of Pharmacy and Pharmaceutical Sciences

Kwame Nkrumah University of Science and Technology

University Post Office

K.N.U.S.T.

Kumasi

Ghana

31^st^ December,2023

The Editor

Editorial Office

PLOS
1265 Battery Street, Suite 200
San Francisco, CA 94111
United States

Dear Editor,

**Letter of rebuttal**

I write to submit the revised manuscript titled: ***Knowledge and prevalence of common sexually transmitted infections among patients seeking care at selected health facilities in Southern Ghana****,* with ID number: PGPH-D-23-01356

I have attached the authors’ responses to the queries from the editors and reviewers.

Counting on your usual cooperation. Thank you.

Yours sincerely

Araba Ata Hutton-Nyameaye

(*MPhil, MPCPharm, PhD*)

**Response to Reviewer Comments raised on the Manuscript:** PGPH-D-23-01356

**Knowledge and prevalence of common sexually transmitted infections among patients seeking care at selected health facilities in Southern Ghana**

Please find below our step-by-step response to the comments raised:

**Journal Requirements:**

1. We do not publish any copyright or trademark symbols that usually accompany proprietary names, eg ©, ®, ™ (e.g. next to drug or reagent names). Please remove all instances of trademark/copyright symbols throughout the text, including ® on page 9.

Response: The trademark name has been removed. Refer to page 8.

**2. In the online submission form, you indicated that "The data is available upon reasonable request". All PLOS journals now require all data underlying the findings described in their manuscript to be freely available to other researchers, either 1. In a public repository, 2. Within the manuscript itself, or 3. Uploaded as supplementary information.**

**This policy applies to all data except where public deposition would breach compliance with the protocol approved by your research ethics board. If your data cannot be made publicly available for ethical or legal reasons (e.g., public availability would compromise patient privacy), please explain your reasons by return email and your exemption request will be escalated to the editor for approval. Your exemption request will be handled independently and will not hold up the peer review process, but will need to be resolved should your manuscript be accepted for publication. One of the Editorial team will then be in touch if there are any issues.**

**Response:** The statement has been updated. The data has been uploaded as a supplementary file. Refer to page 20

**Reviewers' comments:**

**5. Review Comments to the Author**

**Reviewer #1:**

**Data management - data needs to be prepare for reanalysis**

**-Some variables have many subcategories and it is not necessary.**

**A. Age categories: has five categories Less than 21, 21 - 30, 31-40; 41 - 50**

**Greater than 50. I advise the authors to collapse to at least 3 categories for the association to make sense. EG. 18-24, 25-40, 41 and above.**

**B. Level of Knowledge =Poor, Satisfactory, Good, Excellent. Authors can group excellent and good to remain with three categories**

**Response:** The categories has been regrouped as recommended by the reviewer. Refer to pages 9-13

**C. No sexual partners has FIVE categories. I suggest 3 categories no partner, one , two or more**

**Response:** The categories have been regrouped as recommended by the reviewer. Refer to pages 14-15

D. Past relationships has SIX categories. I suggest 3 no relationship, one, two or more

Regrouping categories will improve the results when computing the association and strength of association, especially because some values for subcategories = 1, which dilutes the analysis.

**E. Occupation is also a long list that need regrouping**

**Response:** The above-listed categories have been regrouped. See pages 9-13

**Results**

**When presenting association and strength of association, the authors do not give clear interpretation of the results by subgroup? EG: LINE 171-184 please clarify which SUBCATEGORY is associated with to risk**

**Youth, average age or older age? male or female? smoker on nonsmoker, few or more sexual partners? few or more past relationships, low or high level of knowledge etc. These results will be improved once the subcategories are collapsed as suggested in the data management.**

**Response:** This has been corrected. Refer to page 11

**Discussion**

**For all the statement made in the discussion, the authors cite only one study. This show lack of synthesis and balanced comparison with other studies. I encourage the authors to include more studies because STIs is a global topic and there are a lot of studies even in the region of Africa to compare with.**

**Response:** The discussion has been revised. More studies have been added for a balanced comparison with this current study. Refer to page 13-16

**Abstract**

**After collapse subcategories and running the statistics, the authors have to realign the assignment.**

**Response:** This has been corrected. Refer to page 2

**Reviewer #2: Overall**

**This manuscript presents data from a cross-sectional study among 178 individuals seeking STI care in Ho Municipality in Ghana. Prevalence of chlamydia, gonorrhoea, and syphilis, in this population was 35.3%, 39.2%, and 25.5%, respectively. This is likely to be expected given they are reported to be symptomatic. Multivariate logistic regression is used to assess factors associated with STI diagnosis.**

**The introduction is very broad, and doesn’t provide enough background information to justify the study. If data on current prevalence of STIs in Ghana is available, please provide it. If no such data is available, state this.**

**Response:** The prevalence of STIs in Ghana has been provided. Refer to page 4.

**Furthermore, a large sub-section of the study is based around a single study by Seidu et al., which demonstrated self-reporting of STIs in the previous 12 months from demographic health and surveillance data. The results of the study appear to be misrepresented, and so this section needs to be re-formulated.**

**Response:** This statement has been removed. Refer to page 4

**The methods are relatively clear, and I have just suggested a few minor changes for clarity. However, a significant issue is the lack of symptoms data as it makes it more difficult to determine who the sample is and who they are meant to represent.**

**Response:** The data on symptoms that the patients exhibited has been included in the manuscript. See page 10 (Table 2)

**Some aspects of the results section are unclear, particularly surrounding presentation of data.**

**The discussion needs a significant re-writing. There are several errors related to presenting this study’s own results. Furthermore, some of the comparisons with other studies and data are inappropriate due to differences in study design and populations. There needs to a unifying focus or thread through the discussion – at present, it is quite long but very disjointed, jumping from one topic to the next.**

**Response:** The discussion has been revised. See page 13-16

**Overall, there are several major issues that need to be addressed before being suitable for publication.**

**Abstract**

**Page 2, line 25: “STIs”**

**1. Please provide full term before first use of abbreviations.**

**Response:** The full meaning of the abbreviation has been stated. See page 2

**Page 2, lines 26-28: “This study aimed to determine the knowledge and prevalence of common STIs and associated risk factors among adult patients seeking care in health facilities in Ho Municipality.”**

**2. To ensure the study aim is as accurate as possible, I suggest making it explicit that the sample is adult patients seeking “STI” care.**

**Response:** The study aim has been made explicit by stating that patients used in the study are “adult patients seeking STI care”. See page 2

**3. Please provide numbers alongside the percentages for demographic factors and STI prevalence data.**

**Response:** Numbers have been provided for all percentages for demographic factors and STI prevalence data. See page 2

**Introduction**

**Page 3, lines 50-51: “Sexually Transmitted infections (STIs) are infections acquired through sexual intercourse with an infected partner (1).”**

**4. Please consider re-phrasing or removing the term “infected partner”, as the term “infected” is potentially stigmatising.**

**Response:** The statement has been rephrased to “Sexually Transmitted Infections (STIs) are diseases contracted through sexual activity with a partner who has the infection.” See page 4

**Page 3, lines 51-52: “Globally, STIs affect more than 1 million people worldwide, with one or more infection types occurring per day.”**

**5. I think this sentence is trying to convey that recent data suggests that there are around 1 million new infections of chlamydia, gonorrhoea, trichomoniasis or syphilis per day. This sentence needs to be re-phrased to make this more clear, as currently the meaning is quite confusing. Additionally, specifying the STIs that are being referred to is important. A reference is also required (current reference number 5 would be sufficient). Given that mention of 367 million new cases per year is subsequently discussed in this paragraph, consider removing this sentence altogether.**

**Response:** The sentence has been rephrased for more clarity to “Worldwide, over 1 million people are affected by STIs, with individuals experiencing one or more types of infections each day” and supported by reference number 5. See page 4 (Lines 53-54)

**Page 3, lines 52-53: “Almost 30 different STIs have been established; some of which are easily managed while others are not treatable (2).**

**6. The reference for this statement is a 2013 therapeutics textbook. I suggest providing a more recent and relevant reference. Consider removing the specific number of STIs that have been “established” as this is likely to change and is open to debate.**

**Response:** The sentence has been removed entirely. See page 4

**Page 3, lined 58-60: “For interventions and resources aimed at reducing the burden of STIs to be planned and implemented effectively, the prevalence and incidence have to be quantified (6).”**

**7. The introduction needs to address why this specific study is required. For example, what data is already available on STIs in Ghana? What could then be done with the data? At present, the introduction is very broad.**

**Response:** This has been updated. See page 4

**Page 3, lines 61-62: “In many parts of Africa, STIs are one of the most common reasons for seeking medical attention.”**

**8. Please provide a reference to support this statement**

**Response:** The above statement is referenced. See page 4

**Page 3, lines 65-67: “The prevalence of STI self-reporting was 6% in a study conducted by Seidu et al.(9) in Ghana (9). This means that a small number of patients self-report as compared to the number of patients who are clinically diagnosed with STIs.”**

**9. The comparison drawn is not reflective of the findings of the study by Seidu et al, which looks at the prevalence of STI self-reporting in Ghana demographic and health surveillance data. Men were asked during the survey “if they had contracted a disease through sexual intercourse in the previous 12 months”, to which they answered yes or no. No mention appears to be made in the study, comparing their findings of self-reporting with “patients who are clinically diagnosed with STIs”. Either provide an additional reference to support this comparison or change above. Additionally, the reference number “9” is in two places in the above sentence.**

**Response:** This has been corrected. See page 4

**Page 3, lines 68-71: “Therefore, self-reporting must be encouraged to facilitate the prompt of these infections. Effective public health interventions such as self-reporting are however hinged on understanding the health seeking triggers in persons who may have STIs.”**

**10. Although seeking healthcare when symptomatic should be encouraged, the use of “self-reporting” here is confusing. It appears to be building on the above reference to Seidu et al. But in that study “self-reporting” did not refer to healthcare seeking – it solely referred to whether a participant reported having an STI in the previous 12 months” during a DHS survey. I think this section needs to be re-written with a better representation of what the data in Seidu et al. is presenting. Additionally, I think a word is missing after “prompt”.**

**Response:** This has been updated. Refer to page 4

**Page 3, lines 73-75: “This study therefore sought to identify health seeking behaviours and prevalent STIs as well as knowledge on STIs in persons who were reporting in various health facilities in southern Ghana.”**

**11. As with comment above, please ensure the study aim is as accurate as possible, by making it explicit that the sample is adult patients seeking STI care.**

**Response:** The study aim has been rephrased. See page 4

**Methods**

**Page 6, lines 101-103: “The determination of the sample size was calculated using the Cochran formula, with prevalence assumed to be 7.4% based on a study conducted by Kularatne et al. (11) with a confidence interval of 95%, a precision of 5% and non-response rate of 20%.”**

**12. The referenced study estimated prevalence and trends for STIs in South Africa. Please provide justification for why you felt this was an appropriate comparison, or at least the best available. Additionally, it is unclear how a prevalence of 7.4% was determined from the referenced paper, so please describe how this was calculated.**

**Response:** The sample size was recalculated using a study by Semwogerere et al., 2021. Refer to page 7 (Lines 105-108)

**Page 6, lines 110-111: “Simple random sampling method was used to recruit the patients.”**

**13. Please provide more detail on what is meant by “random sampling method”. Was it truly random sampling, rather than convenience sampling?**

**Response:** The random sampling method has been further elaborated on. See page 7 (Lines 114-117)

**Page 7, line 137: “Also, level of knowledge was assessed as a 24-item composite variable.”**

**14. Please state if these questions are part of a previously validated tool or if the questions were devised specifically for this study.**

**Response:** The questionnaire was developed from existing literature after careful review of the literature hence the questions were devised specifically for this study. Refer to page 7

**15. Please state that as syphilis testing involved only treponemal antibodies, it is unable to differentiate between active vs past infection.**

**Response:** The above suggested statement has been added. See page 8.

**16. With the regression modelling, the factors generally make sense. However, it is important to consider why you think a variable should be included in a model, and its justification. I do wonder whether smoking status is an appropriate variable to take forward into multivariate modelling, as it is likely associated with STI risk for other reasons e.g. socioeconomic status. Furthermore, the number of smokers is very small. If you decide to keep it in, I would recommend a sentence justifying this. Even better would be a conceptual framework for your analysis.**

**Response:** The variables included in this study were added after careful review of literature. Secondly, the variables included in the multivariate analysis were based on the results obtained under the univariate analysis. The variables under were significant under the simple logistic regression were included in the multiple regression. This justifies the reason why the smoking status was included. Refer to the Results section page 9-13.

**Results**

**17. A significant issue is the lack of data on symptoms. Is this available? It is difficult to determine who the sample is meant to represent as patients “with suspected cases of STI” is very broad and heterogenous.**

**Response:** The list of symptoms has been included. Refer to page 6 and 10 (Table 2)

**Page 9, lines 161-162: “The overall prevalence of STIs was 28.65%. Of these 39.22%, 35.29% and 25.49% were gonorrhea, chlamydial infections and syphilis respectively (Table 2).”**

**18. I suggest providing numbers as well as percentages in the main text. Also, it is more useful to provide the individual prevalences also as a percentage of total - it is unusual to provide them as a percentage of total positives.**

**Response:** The numbers have been provided alongside percentages. Refer to page 10

**Page 9, lines 172-173: “The age (p = 0.02), gender (p= 0.01) and level of knowledge (p= 0.04) of the participants were statistically associated with the risk of sexually transmitted infection among the respondents.”**

**19. Please state the specific age category, gender, and level of knowledge that are associated with higher risk of STIs**

**Response:** This has been corrected. Refer to Results section

**20. I recommend providing percentages, odds ratios and 95% confidence intervals in tables 3A and 3B, to quantify the associations between characteristics and STI status more effectively. The Chi2 values have not been provided for all variables – however, these could potentially be removed.**

**Response**: The chi square values have been removed. Refer to the section on Results.

**21. Table 4 – it may be more helpful to provide overall p values for each variable, rather than a p value for each level of each variable.**

**Response:** The odds ratio and confidence intervals have been provided. This has been edited to reflect the reviewers' recommendation. Refer to Table 3 and 4

**22. Table 4 – The “p value (CI)” column is very confusing. Presumably the confidence intervals are for the odds ratios? If so, I suggest an “odds ratio (95% CI) column” and a separate p value column**

**Response:**  The p-values have been removed since the 95% CI can be used to determine significance. Refer to Table 4 and 5.

**Page 12, lines 185-186: “The factors that were found to influence the risk of sexually transmitted infection were the age of the respondent, level of knowledge on STI and gender of the participant.”**

**23. I am assuming this is referring to the multivariate model – if so, please make this distinction clear, for example with a subheading or opening statement.**

**Response:** This has been corrected. Refer to Table 4 and 5

**Page 13, lines 194 – 200: “Health-seeking behavior of participants” paragraph**

**24. Please provide numbers alongside percentages**

**Response:** This has been revised. Numbers have been provided alongside the percentages. Refer to page 13

**Discussion**

**Page 13, lines 203-205: “Evidence from this study showed that one of every four patients who presented with genitourinary symptoms had STI. This burden is of public health importance and is in line with reported estimates of Sub-Saharan Africa accounting for 40% of the world’s burden of STI (12).”**

**25. This is a really odd comparison, drawn between findings from a very specific sub-set of patients in your study, to STI burden across a continent.**

**Response:** The discussion has been revised. Refer to page 13.

**Page 13, lines 205-207: “The burden of STI from this study is about four times the 6% burden reported by Seidu et al.(9) in a research done in Ghana involving self-reported cases among sexually active males.”**

**26. These studies are not comparable. Seidu et al. asked randomly selected individuals in households as part of a DHS survey if they had had STI symptoms in the past year. This study’s sample is based on diagnostic results amongst care seeking individuals with symptoms.**

**Response:** The discussion has been revised. Refer to page 13-14

**Page 13, lines 208-211: “This prevalence of gonorrhoea is inconsistent with the research done by Dela et al.(13), which established that the prevalence of gonorrhea in health facilities within Ghana was 28%. Chlamydia’s prevalence which was reported as 11% by Dela et al.(13) was similar to our study finding.”**

**27. The present study describes a chlamydia prevalence of 35.3% and a gonorrhoea prevalence of 39.2%. I think the gonorrhoea prevalence is therefore more consistent with Dela et al. Whereas the chlamydia prevalence is very different.**

**Response:** The discussion has been revised. Refer to page 13-14

**Page 13, lines 211-213: “Banong-le et al.(14) also stated that the prevalence of syphilis infection was 3.2% in a research conducted in Ghana and this is lower than the findings in this study which proved that the prevalence of syphilis was 7.3% (14).”**

**28. The results section states that prevalence of syphilis was 25.49%, not 7.3%.**

**Response:** The table in the results section has been revised. Thus the prevalence of syphilis is 7.3% of the participants is included in the study. Refer to page 13-14

**Page 13, lines 217-222: “Additionally, the co-infection between gonorrhea and chlamydia was more than half of the co-infected cases. This observation is similar to the findings of Leonard et al.(15) which showed that gonorrhoea and chlamydia have some natural connections which has been depicted by numerical simulation investigations increasing the spread of infection and/or risk of exposure (15). This explanation signifies the reason why the co-infection between the two infections is more prevalent than expected (15) .”**

**29. It is very difficult to draw any conclusions based on this piece of information, given that it is a highly specific sample, particularly with a review article. The conclusion that this is why CT/NG co-infection is more prevalent than expected is therefore not appropriate.**

**Response:** The discussion has been revised. Refer to page 14

**Page 14, lines 225-227: “The risk of contracting an STI amongst the patients aged less than 21 years is high in this study because young adults are often known to be engaged in risky sexual behaviors due to youthful exuberance (16).”**

**30. There are a range of complex factors that contribute to young people being a risk group for STIs. It cannot simply be put down to “youthful exuberance”.**

**Response:** These statements have been removed after the analysis (regrouping of categories) was done again. Refer to page 14

**Page 14/15, lines 240-244: “Females had a reduced risk of contracting STI and this is in with disagreement with the findings of Amu and Adegun.(20) which reported that female teenagers and young women were particularly susceptible to STIs due to the lining of their cervical 242 openings (20). Nonetheless, males are known to have high sexual risk behaviors compared to females and that increased their risk of contracting STI**

**31. The referenced article by Amu and Adegun is focussed on awareness and knowledge of STIs, and does not appear to discuss susceptibility of STIs. This paragraph also raises a more general point for the discussion, whereby a single reference is used to justify a very definitive statement, that potentially ignores other evidence.**

**Response:** The discussion has been revised. Refer to page 14-15

**Page 17, line 290-292: “In addition, this study employed robust analysis such as multiple logistics regression, and considered potential confounders thereby improving on the generalizability and validity of the findings of the study to the population.”**

**32. The study sample was individuals attending for STI care. Even with adjusting for confounders, it is unlikely that these results can be generalised beyond this specific sub-population.**

**Response:** This statement has been removed. Refer to page 15.

**Reviewer #3: Article addressing a topic of interest.**

**The objectives have been clearly defined.**

**The methodology used made it possible to meet the specific objectives.**

**The analysis method used respects statistical principles.**

**The literature review provided a better understanding of the problem highlighted in the article.**

**However, the reasons for choosing the three target health facilities for the study were not specified. However, this information would have helped to better understand the choice of theme.**

**Response:** The three target facilities are the 3 major health providers in the Ho Municipality.

Refer to page 6.
