# Supplementary material for: Knowledge and prevalence of common sexually transmitted infections among patients seeking care at selected health facilities in Southern Ghana
Source: PLOS Glob Public Health. 2024 Jul 1;4(7):e0003422. doi: 10.1371/journal.pgph.0003422 (PMC11216559; doi:10.1371/journal.pgph.0003422)
Supplement: Attachment — Submitted filename: Response to Reviewers.docx [file pgph.0003422.s004.docx]

Response to Reviewer Comments raised on the Manuscript: PGPH-D-23-01356R1

**Knowledge and prevalence of common sexually transmitted infections among patients seeking care at selected health facilities in Southern Ghana**

Review Comments to the Author

Please find below our step-by-step response to the comments raised by the reviewers:

While the previous reviewers’ comments have been addressed, I have still identified a number of items that I believe need to be rectified prior to publication, in particular a discrepancy in the total number of individuals with an STI accounting for co-infections vs the total numbers of individual STIs added together (see comment in results section with asterisks below).

1. Introduction
A. The sentence in lines 53-54 needs clarification to state that 1 million people worldwide acquire a new infection every day, not that 1 million people worldwide are affected by STIs.

Response: This statement has been revised. Refer to page 4 Lines 53-54

B. In line 71, I presume that it is ‘symptoms of STIs’ that are one for the most common reasons for seeking medical attention. If so, please clarify here.

Response: No please, this statement was not in reference to the symptoms of STI.

C. Does the statement ‘knowledge on STIs is limited’ in line 72 refer to individuals’ knowledge of STIs and their symptoms, or knowledge about the epidemiology of STIs in Ghana? Or both? It would be helpful to clarify here, and if it is the latter (epidemiology), to expand on why. E.g. inadequate epidemiologic surveillance, lack of access to etiologic testing, etc.

Response: This statement focuses on individuals’ knowledge of STIs and their symptoms. The statement has been updated. Refer to page 4 Lines 72

2. Methods
A. Line 112 “physician at the various health facilities introduced the study to patients. Who had been diagnosed with STI after consultation” – can you please clarify how STIs are diagnosed normally at the clinical sites. Presumably this is syndromic management but it would be helpful to clarify for readers who may not be familiar with the local standard of care.

Response: The authors have clarified this statement by explaining syndromic management and some clinical syndromes commonly used in diagnosing STIs by the National Standard treatment guidelines. Refer to page 7 Lines 111-117

B. Line 121 – was any of the data collected using the questionnaire qualitative? I believe this should be "quantitative"

Response: This statement has been corrected. Refer to page 7 Line 125

C. Laboratory analysis: the statement in lines 148-150 regarding syphilis testing only involving treponemal antibodies would be more logical to include in the laboratory analysis, rather than the statistical analysis section; recommend moving this up.

Response: This statement has been moved to laboratory analysis section. Refer to page 8 Lines 149-150

D. It would be very helpful to include the list of questions used to assess STI knowledge as part of this manuscript, potentially as a supplementary file, as this would be informative to readers in interpreting the results of the knowledge assessment. Were these questions part of a validated STI knowledge tool used previously in other settings? What do the questions ask about - STI symptoms? transmission? sequelae? How were the score categories of 0-6, 7-12, and 13-24 points to represent low, satisfactory, and good knowledge levels chosen? Is this based on prior literature? Why not divide the participants into tertiles of STI knowledge scores, for example?

Response: The questionnaire is uploaded as a supplementary file. Initial score of categories were 0-6, 7-12, 13-18 and 19-24. However, the first review requested that the category Good and Excellent level of knowledge should be merged. The variable Knowledge has therefore been categorized as tertile with scores 0-8 as poor, 9-16 as satisfactory and 17-24 as good level of knowledge. Refer to page 9 Lines 161-163

E. Please provide additional details regarding the measures collected in the questionnaire (and presented later in Table 4). For example, does “sexual partners” mean number of sexual partners in a specific time frame (eg, past 1 month?). What does “past relationships” mean – is this number of prior lifetime sexual partners or something else? Is condom use and sexual intercourse per week only asked of/reported for participants who report a current/recent sexual partner, or everyone? How is “heard of STI” asked or assessed? Is alcohol use any alcohol use at all, or over a certain amount, and in what time frame? Consider including a copy of the questionnaire as a supplementary file.

Response: Further explanations have been made to the terms used in this study. Refer to pages 7 and 8 Lines 127-132

F. Line 160 – was a p-value threshold of <0.05 from the bivariate analyses used to determine if a variable would be included in the multivariable logistic regression, or a different threshold?

Response: Yes ,a p-value threshold of <0.05 from the bivariate analyses was used to determine if a variable would be included in the multivariable logistic regression.

3. Results
A. Line 170 – Please define the acronym OPD for readers who may not be familiar with this.

Response: The acronym has been defined. Refer to page 10 Lines 178

B. Table 2 – Consider simplifying the table by removing or combining the cells for symptoms that are not applicable to one of the genders (eg, discharge from penis for female participants)

Response: The table has been simplified as suggested. Refer to page 10 Table 2.

C. Lines 180-181 – The statement “low levels of knowledge did not translate to higher number of infections” seems like a conclusion to be made from a statistical analysis demonstrating that there was no association between level of knowledge and STI prevalence; it would thus be clearer to make this statement later in the results section where the univariate analyses are presented.

Response: Though the authors acknowledge the reviewer’s comment, the authors consider it important to keep the statement since Table 3 indicates the relationship between knowledge of STI and prevalence of STI.

D. **Table 3 and lines 179-184 - Since there were 7 participants with co-infections, the total number of individuals with any STI (one or more) cannot be the same as the sum of the individual STIs. In looking at the number and type of coinfections described in lines 181-184, I have calculated out the total number of individuals with any STI to be 42 rather than 51, giving a different overall prevalence of any STI in the sample (42/178 = 23.6%). Please confirm if this is correct and amend the results accordingly. Additionally, note should be made in table 3 that individuals with co-infections are being counted multiple times in the table.

Response: The data has been reanalyzed hence all the figures have been amended. Refer to Table 3.

E. Line 188 – Age does not show a statistically significant association with having an STI based on table 4, instead, smoking status and number of past relationships do and should be included in this sentence instead of age.

Response: This statement has been rephrased. Refer to page 12 Lines 197-198

F. Line 211 – please define ‘orthodox treatment’

Response: This term has been defined. Refer to page 8 Lines 132-133

4. Discussion
A. Line 219-220: It is unclear why the authors have compared the STI prevalence found in this study with STI prevalence among a different population (pregnant women) in a different part of the world (Nepal). This is not a relevant comparison, as there is no reason to suspect that these two prevalence rates should be similar. There are many studies reporting STI prevalence in more similar populations (symptomatic adults of both genders in West Africa) that would be a more appropriate comparison to the present study.

Response: The authors have cited similar studies (reference 20,21,22). Refer to Page 14 Lines 226-230

B. Lines 222 – 223: these percentages represent percent of the total cohort, not percent of the total infections.

Response: This statement has been rephrased. Refer to page 14 Lines 231.

C. Lines 223 – 229: were the Banong-le et al and Nyarko et al studies conducted among symptomatic patients or those attending STI services? Would be helpful to mention this if the prevalence rates are being compared.

Response: Both studies were conducted among symptomatic patients hence clarified. Refer to page 14 and 15 Lines 232-239

D. Lines 236-238: is diagnostic testing available in Ghana? While increasing diagnostic testing would help avoid both over- and under-treatment of STIs, this is not available in many parts of the world. An acknowledgement and/or discussion of this challenge would strengthen the discussion section overall.

Response: A brief discussion on the challenges involving the use of diagnostic testing has been included. Refer to page 14 Lines 244-250

E. Lines 240-243: There is an important opportunity here to discuss the challenges faced by individuals in preventing STIs despite high levels of knowledge. Many factors have been identified, including social norms that discourage condom use, gender power imbalance, inability for women to negotiate condom use/safe sex, etc.

Response: The authors have updated the discussion with the challenges faced by individuals in preventing STIs despite high levels of knowledge. Refer to Page 15 Lines 254-260

F. Lines 249-250: It would be helpful to mention that there were only 7 smokers total in the whole cohort and that the 95% confidence interval for the odds of STI for smokers vs non-smokers was very wide in multivariable analysis (1.07-39.56), therefore, these results have to be interpreted with caution, and it is possible that the risk of STI is only slightly higher for smokers vs non-smokers.

Response: This statement has been updated. Refer to page 16 Lines 268-271.

G. Lines 250-254: As above, I would be cautious with interpretation of this finding and with the results of the literature. In the Berg et al study, smoking was “associated with” higher risk sexual behaviors, it cannot be concluded that smoking itself “enhanced” a person’s tendency for higher risk sexual behavior.

Response: The statement has been rephrased. Refer to page 16 Lines 273-275

H. Lines 261-262: have any studies (behavioral science, qualitative studies) reported on the cultural sensitiveness of discussing genitourinary issues in the Ghanaian setting? It would be helpful to cite literature from Ghana or a similar setting to support this statement.

Response: The authors have cited a study in Ghana (reference 30). Refer to page 16 Lines 282-283

I. Lines 263-264: did the study participants actually report that they had low confidence in the overall health system? If not, I would be clear here that this is only a possible explanation for the observed delays in seeking care.

Response: The questionnaire did not include questions on health system confidence hence the explanation for the observed delays in health-seeking behavior was based on an assumption.

J. Lines 278-279: the statement regarding incorporation of herbal medicines into the national treatment guidelines, while important, seems contradictory to the point made in the previous sentence regarding lack of evidence on efficacy and safety of herbal medicines. Perhaps the need is for greater research on the safety and efficacy of herbal medicines so that those that are safe and efficacious could be incorporated into treatment guidelines.

Response: This statement has been rephrased. Refer to page 17 Lines 298-301

Conclusion

Line 295 – as only associations can be drawn, I would state that marital status and smoking “were associated with” (rather than “influenced”) STI risk

Response: This statement has been rephrased. Refer to page 18 Lines 317-319.

As STI knowledge was a major focus of the study, I would consider stating the finding that low STI knowledge was not associated with having an STI in the conclusions section.

Response: This statement has been added. Refer to page 18 Lines 316-317

Reviewer #5: The Authors have sufficiently addressed the concerns raised by the previous reviewers
