# Supplementary material for: Knowledge and prevalence of common sexually transmitted infections among patients seeking care at selected health facilities in Southern Ghana
Source: PLOS Glob Public Health. 2024 Jul 1;4(7):e0003422. doi: 10.1371/journal.pgph.0003422 (PMC11216559; doi:10.1371/journal.pgph.0003422)
Supplement: Attachment — Submitted filename: Response to Reviewers.docx [file pgph.0003422.s005.docx]

Response to Editor’s comments raised on the Manuscript: PGPH-D-23-01356R2

**Knowledge and prevalence of common sexually transmitted infections among patients seeking care at selected health facilities in Southern Ghana**

Editor’s Comments to the Author

Please find below our step-by-step response to the comments raised by the editors:

A. Journal Requirements:

Please review your reference list to ensure that it is complete and correct. If you have cited papers that have been retracted, please include the rationale for doing so in the manuscript text, or remove these references and replace them with relevant current references. Any changes to the reference list should be mentioned in the rebuttal letter that accompanies your revised manuscript. If you need to cite a retracted article, indicate the article’s retracted status in the References list and also include a citation and full reference for the retraction notice.

Response: Reference list is complete and correct.

B. Additional Editor Comments (if provided):

1) The prevalence rates reported in the results do not reflect the true prevalence of each STI, as those STIs that were part of co-infections are not counted. Unless there is a reason to treat STIs that are detected as monoinfections vs co-infections differently (this would be unlikely), recommend that the prevelance rate for each STI is reported as the total number of people with each STI (including those with mono-infections and those with co-infections) dividided by the total sample size. of note, the sum of these prevalence rates will not be the same as the prevalence rate for any STI, as people with coinfections will only be counted once for the overall STI rate.

Response: The prevalence of various STIs has been corrected to include the cases of co-infections. Please refer to Page 2 Lines 38-39 and Page 14 Lines 233-235

2) Methods - clarify in lines 128-129 whether past relationships were lifetime relationships or relationships in past year.

Response: The variable labelled ‘Past relationship’ measures the number of past relationships a respondent has in the past year. This has been corrected to reflect this explanation in the manuscript. Refer to Page 8 lines 128 -129

3) Specify which answers on the STI knowledge survey were considered 'correct' (in the supplemental data)

Response: Correct responses to the items on the STI knowledge are specified on the supplemental data (questionnaire) as suggested.

4) Add to limitations:

-Non-validated STI knowledge assessment used, may not be generalizable

-Difficult to make associations between STI knowledge and STI prevalence among those already symptomatic - even those with low knowledge are arriving bc they’re seeking care. There could still be an association between STI knowledge and STI prevalence among people with asymptomatic infections.

Response: These have been added to the limitations. Refer to Page 18 lines 314 - 318
